# Selections

**Published:** 1862-09

**Authors:** 


### SELECTIONS.

The Editor of the *Boston Medical Journal* makes the following remarks in regard to "Surgeons for the New Levy:" "The sudden call to arms of six hundred thousand men brings with it a demand for the services of the medical profession, which it needs all the stimulus of patriotism to meet. Six hundred thousand men will, under the present laws, require eighteen hundred medical men to fill the offices of regimental and assistant surgeons. The fact is somewhat startling to contemplate, and it is important that our brethren should be preparing their minds for the responsibility which it implies. In this State alone over a hundred surgeons will be required to fill the new regiments. We may well ask, where are so many fit men to be found? As a general rule, thus far Massachusetts has done well in its appointments on the medical staff. A careful preliminary examination has had the effect of sifting out very thoroughly the chaff from the wheat, and the urgent need of the time has called into the field some of the best men of the profession. We sincerely hope that the same care will be exercised now, and that the exigency of the moment will not lead to the appointment of men unworthy of the place. Above all, there should be no favoritism; no commission given on personal grounds alone. Such appointments are more than likely to fall upon those who could get them in no other way, and who are therefore the last persons who should have them. Of course it must be a matter of extreme difficulty to find so many men as are wanted who are practically familiar with the most important operations of surgery. This is a serious evil, as novices are only too ready to signalize themselves by daring operations. An extract which we printed last week fully confirms this statement. Operative surgery in reality, as the experience of the past year has proved, constitutes the smallest part of the duty of an army surgeon; his chief duty is to treat disease, and dress rather than make wounds. Such being the fact, there are hundreds of men in Massachusetts competent at this moment to assume the responsibilities which belong to the office. There will always be some among those who obtain appointments, whose special tastes and previous experience fit them particularly for the graver operations likely to be necessary. This fact must come out in the course of their examination, or through the personal knowledge of the State medical commission, and upon such men the duty should be put. The mind shudders at the thought of an amputation taking *an hour and a half* in its accomplishment, commenced by the flap operation and ending by circular incision, such as we are credibly informed has, at least in one instance, within a year, been done by the bungling hands of a novice, whose official position should have been no warrant for such an outrage. As the post of brigade-surgeon has been recently abolished, we know of no way, unless it be through the medical director of a division, by which the responsibility of doing capital operations may be assigned by any medical officer above the rank of regimental surgeon to the men especially fit. If such authority does exist, we sincerely hope it may be exercised without any undue delicacy. We trust that the spirit which leads so many of our brethren to give up their practice at home, to incur the dangers and the labors of military life, will lead them to abstain from making their position an excuse for undertaking what they ought not to perform, if more competent men are at hand.

With regard to assistant surgeons, we see no reason why, in the present emergency, medical students in the last year of their course may not receive the appointment. The position is subordinate, and does not call for any professional knowledge which is not at the command of many we could name, whose youth and physical vigor also admirably qualify them for the arduous labor which its duties impose."

